# Systemic Lupus Erythematosus and Cardiovascular Diseases: A Systematic Review

**DOI:** 10.7759/cureus.39284

**Published:** 2023-05-21

**Authors:** Mohammed A Nor, Oboseh J Ogedegbe, Ahmed Barbarawi, Abdirazak I Ali, Ibrahimkhalil M Sheikh, Feisal M Yussuf, Siad Mohammed Adam, Omar A Hassan, Godfrey Tabowei, Abdulmalik Jimoh, Eunice O Mejulu, Asfand Yar Cheema

**Affiliations:** 1 Internal Medicine, Stamford Hospital/Columbia University Vagelos College of Physicians and Surgeons for Internal Medicine, Stamford City, USA; 2 Internal Medicine, Lifeway Medical Center, Abuja, NGA; 3 Internal Medicine, University of Minnesota, Minneapolis, USA; 4 Pediatrics, University of Minnesota, Minneapolis, USA; 5 General Practice, Erciyes University, Rochester, USA; 6 General Practice, Antaliya Hospital, Garissa, KEN; 7 General Practice, Baskent University Faculty of Medicine, Adana, TUR; 8 General Practice, Ondokuz Mayis University, Samsun, TUR; 9 Internal Medicine, California Institute of Behavioral Neurosciences & Psychology, Fairfield, USA; 10 Internal Medicine, Mount Horeb Clinic and Dialysis Center, Warri, NGA; 11 Public Health, Western Illinois University, Macomb, USA; 12 Medicine, Services Hospital, Lahore, PAK; 13 Internal Medicine, Lahore Medical and Dental College, Lahore, PAK

**Keywords:** libman sacks endocarditis, atherosclerosis, neonatal lupus, lupus myocarditis, cardiac tamponade, pericarditis, cardiovascular diseases, systematic lupus erythematosus

## Abstract

Systemic lupus erythematosus (SLE) is an autoimmune condition characterized by multi-organ involvement. The clinical presentation often varies from mild to moderate to severe. The cardiovascular system may also be affected, often portending a poor prognosis for patients. Although the relationship between SLE and cardiovascular disorders has been extensively explored through case reports and literature reviews, few systematic reviews explicitly focusing on this association have been conducted. In light of this, this systematic review aims to analyze the extent of the association between SLE and cardiovascular diseases (CVDs), by exploring the risk of developing CVDs, including myocardial infarction (MI), atherosclerosis, myocarditis, pericarditis and arrhythmias, in SLE patients vs. non-SLE patients. We followed the Preferred Reporting Items for Systematic Reviews and Meta-Analyses (PRISMA) guidelines to perform the systematic review. A detailed search was done covering the period from March 2003 to March 2023 using three databases: PubMed, Google Scholar, and Cochrane. The PubMed search identified 597 articles, while Google Scholar and Cochrane searches yielded 559 and three articles, respectively.

Of the 1159 articles retrieved, we chose eight for final consideration, after excluding papers that did not discuss the role of SLE in CVDs, papers published earlier than 2003, and papers with incomplete data. The eight studies chosen included two narrative reviews, two systematic reviews, and four observational studies. In this systematic review, SLE was proven to have a strong relationship with diverse CVDs, including rare ones scarcely discussed in the literature, such as vasculitis and aortic dissection. All eight of the final papers indicated a connection between SLE and CVDs, based on the systematic analysis of these articles, which revealed that most recent research supports a higher risk of peripheral arterial occlusive disease (PAOD), MI, pericarditis, myocarditis, and other cardiovascular disorders in individuals with SLE. These associations may have certain gray areas, as patient characteristics and comorbidities often affect the extent of illness and long-term prognosis. Larger-scale studies are required to probe this relationship further and research the etiopathogenesis involved in order to improve patient outcomes. The effects of SLE on the heart are, however, unequivocal.

## Introduction and background

The classical autoimmune disease, systemic lupus erythematosus (SLE), is associated with a significant global disease burden. However, epidemiological estimates differ considerably between various ethnic, racial, and age groups [1]. The condition is more prevalent in younger women, with a female-to-male prevalence ratio of approximately 10:1 [2]. Survival rates in SLE have significantly improved, yet deaths due to cardiovascular disease (CVD) in lupus have not [3]. CVD accounts for approximately one-third of deaths in SLE and, in some cohorts, is the leading cause of mortality in lupus [4]. This illness is characterized by numerous clinical manifestations, antibodies, and the involvement of one or more organ systems [5]. These antibodies produced in SLE are directed to a host of self-molecules found in the nucleus, cytoplasm, and cell surfaces [6]. These include antinuclear antibodies (ANAs) (present in more than 95% of patients), anti-double stranded DNA (ds-DNA), and anti-Smith (ant-Sm antibodies).

The main histopathological features of SLE are inflammation and blood vessel abnormalities, band or occlusive vasculopathy, vasculitis, and immune complex deposition. Several factors are responsible for the loss of immunological tolerance against self-antigens in this condition. These factors are environmental, endocrine, genetic, and immunological, resulting in the development of autoantibodies that cause tissue damage through diverse mechanisms [7]. This pathological mechanism is explained as an interruption of tolerance in genetically predisposed people and exposure to environmental factors, causing the activation of autoimmunity [7]. Cardiac involvement in patients with SLE can negatively impact all components of the cardiovascular system and heart, including the pericardium, conducting system, myocardium, valves, and coronary arteries, and is associated with increased morbidity and mortality [8].

Cardiovascular manifestations in SLE result from numerous pathophysiological mechanisms that work in tandem, and increase morbidity and mortality in SLE patients [2]. The malfunction of endothelial cell activation, which results in the development of lectin-like oxidized low-density lipoprotein receptor 1 (LOX-1), causes endothelial cell dysfunction [2]. By causing the release of tissue necrotic factor-alpha (TNF-a), interleukin 6 (IL-6), and interleukin 12 (IL-12), which are essential for attracting monocytes to adhere to endothelial cells, LOX-1, a pro-inflammatory receptor, raises the risk of CVDs in SLE [9]. Another mechanism of cardiac disease in SLE is the disruption of innate immunity [2]. This immune dysregulation is associated with a distinct subset of lupus pro-inflammatory neutrophils known as low-density granulocytes (LDG), which are found in increased numbers in SLE and promote in vitro endothelial cell damage [10]. These LDGs trigger the formation of neutrophil extracellular traps (NETs), which facilitate unstable coronary plaque and thrombus formation [11]. A third mechanism of cardiac disease in SLE is the disruption of adaptive innate immunity [2]. This occurs through the excessive activation of T lymphocytes, including CD4+ T cells, promoting vascular injury and thrombus formation by IFN-1 signaling [12]. This systematic review aims to examine and consolidate the relevant information on the connection between SLE and CVDs.

## Review

Methods

The Preferred Reporting Items for Systematic Reviews and Meta-Analyses (PRISMA) criteria were used to perform a systematic literature search [13].

Eligibility Criteria

We employed specific inclusion criteria to select the studies relevant to our systematic review. Studies evaluating the role of SLE in the etiopathogenesis of various cardiovascular diseases were selected as the primary target of our research. We decided to gather studies published from 2003 to 2023, including free full texts, systematic reviews, traditional reviews, case reports, literature reviews, and observational studies. Studies on animals, those with incomplete data, those with free full-texts unavailable, and articles that did not meet our research goals were excluded.

Selection Strategy

Two authors conducted a thorough screening (M.A.N. and A.B.), each blinded to the other's ratings, employing identical search plans in all databases, and wherever they disagreed about the articles' fulfillment of the inclusion or exclusion criteria, a third author (I.M.S.) intervened. Initially, articles were reviewed using titles and abstracts, and afterward, the entire articles were read. The search was conducted by three authors (I.M.S, O.A.H, and S.M.A) between March 12, 2023, and April 2, 2023.

Database and Search Strategy

To conduct this systematic review, we selected studies from PubMed, Google Scholar, and Cochrane Database, covering the period from March 2003 to March 2023, to elicit only the most relevant articles for our systematic review assessing the role of SLE in the etiopathogenesis of CVDs. Keywords used in all search engines were as follows: 'SLE' or Systemic Lupus Erythematosus' AND 'cardiovascular diseases' or 'cardiac diseases' or 'heart diseases' or 'cardiac manifestations'. We combined the keywords in every combination to generate the maximum number of articles for screening.

Analysis of Study Quality

Out of the eight articles selected for this study, two were narrative reviews, two were systematic reviews, and four were observational studies. The quality assessment tools used for this systematic review included the Newcastle Ottawa Scale (NOS) [14] for cohort and case-control studies; Assessment of Multiple Systematic Reviews 2 (PRISMA 2020 Checklist) for systematic reviews and meta-analyses [13]; and Scale for the Assessment of Narrative Review Articles (SANRA) for narrative reviews [15]. Each assessment tool had its own criteria and different systems of scoring. Two reviewers, independently using commonly used tools for each type of study, assessed each selected study for risk of bias. We accepted a score of at least 70% for each assessment tool to ensure that only high-quality papers were used. Table 1 gives further insight into the quality assessment tools and the accepted articles.

**Table 1:** Quality assessment of each study PRISMA: Preferred Reporting Items for Systematic Reviews and Meta-analyses; SANRA: Scale for the Assessment of Narrative Review Articles

Quality Assessment Tool	Type of Study	Total Score	Accepted Score (>70)	Accepted Studies
PRISMA [13]	Systematic review and meta-analysis	44	82%	Bello et al. [16], Yazdany et al. [17]
Newcastle Ottawa Scale [14]	Cohort and control studies	8	75-88%	Chuang et al. [18], Gao et al. [19], van der Laan-Baalbergen et al. [20], Zhang et al. [21]
SANRA [15]	Narrative reviews	12	75-92%	Alghareeb et al. [2], Frostegård [22]

Results

**Figure 1:**
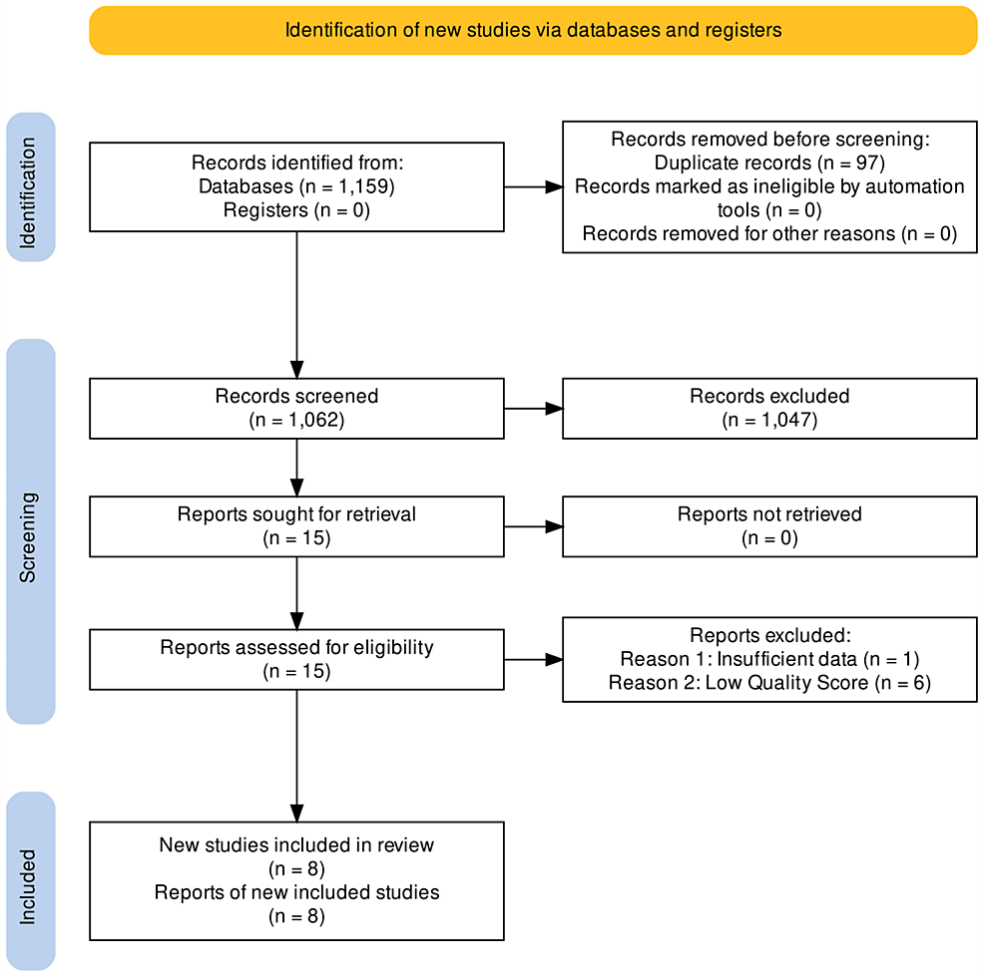
PRISMA flowchart of the study selection process PRISMA: Preferred Reporting Items for Systematic Reviews and Meta-analyses

The comprehensive and exhaustive database searches of PubMed, Google Scholar, and Cochrane elicited 1159 potentially related titles based on the study inclusion criteria for this systematic review. The removal of duplicates left 1062 articles, after which all remaining records were screened using the title and abstract. A secondary review was then carried out by reading full-text articles and using detailed inclusion and exclusion criteria to eliminate irrelevant articles not possessing the required data for this systematic review. This holistic evaluation yielded 15 articles related to our research question, which were further subjected to the screening tools (PRISMA, SANRA, and Newcastle Ottawa Scale). After a quality appraisal, we eliminated seven studies, and the remaining eight articles were included in our systematic review. These articles comprised two systematic reviews, four observational studies, and two narrative reviews. We created a PRISMA flowchart for study identification and filtering of the articles as shown in Figure 1 [13]. Table 2 summarizes the features of the articles included in the review.

**Table 2:** Characteristics of papers included in the study CVDs: cardiovascular diseases; SLE: systemic lupus erythematosus; MA: meta-analysis; RR: relative risk; AR: attributable risk; IR: incidence rate; MI: myocardial infarction; HR: hazard ratio; CI: confidence interval; ANA: antinuclear antibodies; T2DM: type 2 diabetes mellitus; HF: heart failure; VTE: venous thromboembolism; LM: lupus myocarditis; SLEDAI: Systemic Lupus Erythematosus Disease Activity Index; NHIRD: National Health Insurance Research Database; PAOD: peripheral arterial occlusive disease; ACR: American College of Rheumatology

Author	Year of Study	Type of Study	Methods	Limitations	Conclusions
Alghareeb et al. [2]	2022	Narrative review	Doppler echocardiography was used to evaluate the patients	The study, which primarily focused on the pathophysiology and clinical manifestations of CVD linked with SLE, did not discuss the diagnostic tools and screening procedures for CVDs	The clinical significance of this review article is to show how SLE and CVDs, such as premature atherosclerosis and the ensuing acute coronary syndrome, heart failure and stroke, and carditis, which affects various heart layers and heart valves, are intercalated. Even when the patient is in remission, these deadly consequences lead to repeated hospitalizations and lower life expectancy and quality of life
Bello et al. [16]	2023	Systematic review	A random effects model was used to produce the pooled risk estimates. Three studies provided the corresponding measure for each outcome, and meta-analyses for RR, AR (incidence proportion), and IR were done. The proportion of variance explained by heterogeneity was calculated using Higgins's I^2^. R (R Foundation for Statistical Computing, Vienna, Austria) was used for the analyses, namely metaprop or metafor. Visual inspections of forest plots were conducted to evaluate the consistency of the study's results.	All observational studies have the potential for bias, which is a drawback. Moreover, the inclusion of various study types probably played a role in the variability seen in the majority of endpoints	Data from this MA indicate that patients with SLE had a higher risk and incidence of stroke, MI, and CVD than people without SLE. It is crucial that risk variables are frequently identified and suitable preventive strategies are included in the management plan given the significant risk of CV mortality and morbidity in patients with SLE, especially in younger patients
Chuang et al. [18]	2015	Observational study	After using the Kaplan-Meier method to create the PAOD cumulative-incidence curves for the SLE and control groups, the log-rank test assesses the discrepancy between the two incidence curves. A single-variable and multivariable Cox proportional hazard models were used to determine the HRs and 95% CIs to assess the PAOD risk in SLE patients	The laboratory results for each SLE patient cannot be made available by the NHIRD (such as disease activity in SLE and autoantibody of lupus anticoagulant). As a result, further research could not be conducted into the possible connection between peripheral artery occlusive disease and lupus anticoagulants. Secondly, there might have been an overestimation of the prevalence of PAOD patients who showed no symptoms, or only mild symptoms might have been ignored. Finally, information from the NHIRD regarding cardiovascular risk factors, such as smoking, eating habits, body mass index, and the level of daily activity, were not found	People with SLE have a higher risk of PAOD, and SLE is a risk factor for PAOD. Paying attention to the risk of PAOD among young SLE patients is essential as this risk is significantly higher. More studies on illness detection and early intervention are needed to stop PAOD issues in SLE patients
Frostegård [22]	2023	Narrative review	The development of SLE diagnostic criteria is fascinating in and of itself. The most recent diagnostic criteria were published in 2019 by the European League Against Rheumatism/American College of Rheumatology, where positive ANAs were required as an entry criterion, and a combination of clinical, serological/immunological manifestations and measures served as the basis for diagnosis	Though more recent studies on the advantages of statin use have been published, there are few studies on the use of statins for SLE	Due to increased thrombosis risk and increased atherosclerosis, particularly atherosclerotic plaques, SLE patients have a greater risk of developing CVD. This poses a significant clinical issue and may provide insight into atherosclerosis's inflammatory and immunological underpinnings. This elevated risk is caused by a mix of conventional and novel risk factors, including disease activity linked to SLE. Traditional risk factors should be tackled, and SLE treatment should be optimized to prevent and manage CVD in SLE
Gao et al. [19]	2022	Observational study	The most recent diagnostic criteria for SLE were published in 2019 by the European League Against Rheumatism/American College of Rheumatology, where positive ANAs were required as an entry criterion, and a combination of clinical, serological/immunological manifestations and measures served as the basis for diagnosis	Ethnicity affects the prevalence and mortality of SLE. It is more challenging to explain the probable causal link between SLE and CVD in other populations because all individuals in this MR analysis were Europeans. Furthermore, the OR value was relatively low and must be carefully evaluated	The findings of this investigation support the hypothesis that SLE may raise the risk of HF and VTE while decreasing the risk of T2DM. This study will advance our comprehension of the fundamental illness mechanisms underlying SLE and offer complete CVD diagnosis and care for SLE patients. We anticipate more studies aimed at lowering CVD morbidity and mortality in SLE patients. Given the size of the causal influence, the MR estimations in this study should be regarded with care
van der Laan-Baalbergen et al. [20]	2009	Observational study	A retrospective case analysis of SLE patients from two tertiary referral facilities who initially manifested cardiac involvement between 1999 and 2004 with clinical and echocardiographic symptoms of heart failure was performed. The clinical presentation, subsequent care, and serial echocardiography results were all recorded.		Heart failure is a rare but potentially fatal symptom of cardiac involvement in SLE. The long-term results can be great when active therapy is started right away. Myocarditis-related heart failure can present as SLE's cardiac manifestation, necessitating immediate treatment. Using echocardiography as a diagnostic tool is crucial
Yazdany et al. [17]	2020	Systematic review	If studies offered estimates of the effect sizes that could be used to produce pooled effect estimates, those studies were taken into consideration. Random-effects models were used to calculate the pooled risk ratios and 95% CIs for stroke and MI. The sensitivity analyses investigated bias, while the I^2^ test assessed heterogeneity	Heterogeneity was found among the assessed studies, which may be due to differences in demographic characteristics, the choice of the control group, and the reported risk measure. The degree to which the SLE and comparison populations were matched for CVD risk variables could be another source of variability	Adult patients with SLE have a two- to three-fold higher risk of stroke and MI than the general population or healthy controls. The observed higher risk is most likely caused by known risk factors for MI, stroke, and SLE. Understanding the multiple processes driving elevated CVD risk in SLE patients, particularly how antiphospholipid syndrome or antibodies to phospholipids may modulate this risk, will promote prevention and therapy methods and enhance well-informed patient and clinician decisions
Zhang et al. [21]	2015	Observational study	Univariable analysis was carried out using Chi-squared tests for categorical variables and the student's t-test or Mann-Whitney U-test for continuous variables depending on the normality. One hundred individuals with SLE but without LM were randomly pooled as the control group, whereas 25 patients with LM from 2001 to 2012 were included as the study group	Most patients with LM did not have access to coronary angiograms or myocardial biopsies to rule out cardiomyopathy caused by other causes, one of the study's significant weaknesses. An expert attending cardiologist had performed a strict differential diagnosis based on the clinical data, a frequent diagnosis technique in actual practice	LM is a rare but significant organic involvement of SLE. The majority of LM happens towards the beginning of SLE. The primary independent risk factor for LM was a high SLEDAI score. The diagnosis may be supported by characteristic echocardiographic findings consistent with LM. Even though LM rarely causes death, the results are typically positive after intensive immunosuppressive medication

Discussion

In a cross-sectional study by Mohamed et al. involving 59 SLE patients without any clinically discernible heart disease, numerous echocardiographic abnormalities were found. These included valvular lesions (47.5%), pericardial effusion (13.6%), pulmonary artery hypertension (8.5%), pericardial thickening (6.8%), impaired systolic function (3.4%), and left ventricular hypokinesia (1.7%) [23]. Also, no abnormality was seen in 44.1% of the patients [23].

Pericarditis is the most common cardiac manifestation of SLE [24]. However, it is uncommon for acute pericarditis to be the leading symptom at the time of diagnosis of SLE, and it occurs in just 1% of patients [25]. A case report by Bezwada et al. discussed myopericarditis with pericardial effusion as the initial presentation of SLE [26]. Furthermore, Gupta et al. published a case report on constrictive pericarditis as the initial manifestation of SLE [27]. In this case, echocardiography showed a thickened pericardium with effusion and motion abnormalities consistent with constrictive pericarditis. At the same time, a CT scan of the chest revealed pericardial effusion, pericardial thickening, and normal lung parenchyma with mild bilateral pleural effusion. Narang et al. published a case series discussing three patients who developed acute pericarditis without prior history of SLE but were later diagnosed with SLE [25]. On investigation, two of these cases had the classical electrocardiography finding of diffuse ST-segment elevation with PR interval depression. At the same time, one demonstrated low voltage without signs of PR and ST-segment changes and electrical alternans. Non-steroidal anti-inflammatory drugs (NSAIDs) are the primary choice in managing pericarditis; however, SLE treatment includes disease-modifying anti-rheumatologic drugs (DMARDs), glucocorticoids, and even immunomodulators as SLE is an organ-threatening condition [25].

A systematic review and meta-analysis by Bello et al. sought to evaluate the relative risk of cardiovascular disease outcomes in SLE patients vs. the general population. They concluded that there is a statistically significantly higher risk of stroke in those with SLE, with the relative risk ranging from 1.61 to 5.34 and a pooled estimate of 2.51 (95% Cl: 2.03-3.10) [16]. In addition, this study found a 2.9-fold statistically significant higher risk of myocardial infarction (MI) in patients with SLE [RR: 2.9 (95% Cl: 2.45-3.48)] [16]. Furthermore, the RR of hypertension was also statistically significant compared to the general population [RR: 2.7 (95% CI: 1.48-4.92)]. These findings were endorsed by Yazdany et al., whose study reported statistically significant higher relative risks for stroke [2.13 (95% CI: 1.73-2.61)] and MI [2.99 (95% CI: 2.34-3.82)] [17].

Cardiac tamponade is, however, a rare entity, estimated to occur in fewer than 1% of patients with SLE [28]. Emorinken et al. discussed a case of cardiac tamponade as an unusual first presentation of SLE. Findings, in this case, included the chest radiograph showing an increased cardiac silhouette, an expected result in cardiac tamponade [29]. Investigations showed a massive proximate pericardial effusion measuring 2.7 cm in the parasternal long-axis view and 2.4 cm in the subcoastal view [29]. Additionally, ANAs with a titer of 1:1280 (<1:80), anti-Sm, anti-SSA, and anti-dsDNA antibodies were all positive [29]. Chourabi et al. have reported that when cardiac tamponade is diagnosed with SLE, it often presents as the initial finding in patients with a previously undiagnosed disease [30]. Goswami et al. conducted a case series involving 409 patients with SLE. They reported that pleuritis, the size of the effusion, and the presence of anti-nucleosome antibodies were essential predictors of tamponade in the 24 patients who developed tamponade [31].

Zhang et al. conducted a retrospective case-control study on 125 lupus myocarditis (LM) cases [21]. The cases included 25 patients with SLE who developed LM, while the controls comprised 100 of those who had SLE but did not develop LM. In seven cases (28%), LM was the first presentation of SLE and presented at earlier stages, which was in direct contrast with the controls (20.88 ± 35.73 vs. 44.08 ± 61.56 months, p=0.008) [21]. Furthermore, 23 of the LM cases (92%) showed reduced left ventricular ejection fraction (<50%), and all patients had wall motion abnormalities [21]. Studies have shown that LM is not a common manifestation of SLE, presenting in about 9% of SLE patients [32].

A case series of eight cases of neonatal lupus by Teixeria et al. discussed the presence of positive autoantibodies anti-Ro/SSA (90% of the cases), anti-La/SSB, and anti-U1 RNP4 in mothers of affected infants [33]. All mothers were diagnosed with SLE/antiphospholipid syndrome, and seven out of the eight cases developed cardiac manifestations of neonatal lupus, with five having complete heart block and the other two having second-degree heart block and bradycardia, respectively [33]. Neonatal lupus as a disease entity occurs due to passively-acquired autoimmunity, in which tissue injury in the fetus results from the transplacental transfer of maternal IgG autoantibodies to SSA/Ro and SSB/La intracellular proteins [34]. These maternal antibodies cross the placenta to fetal tissue as early as the 11th week of gestation [35]. Skin, liver, and blood cells are regenerative; hence, the effect of these antibodies is short-lived. However, cardiac cells are not regenerative, and therefore, permanent reversal of third-degree heart block has never been seen in the literature [34]. CHB carries significant mortality (20-30%, primarily fetal/ neonatal) and morbidity (67% require permanent pacing before adulthood) [36].

Studies have shown that SLE patients are at increased risk of accelerated atherosclerosis, a notable cause of morbidity and mortality in these patients. A cohort study by Chuang et al. sought to holistically define the association between SLE and peripheral arterial occlusive disease (PAOD); 10,144 SLE patients and 10,144 controls were enrolled in the study [18]. In the SLE group, compared to the non-SLE cohort, the incidence of PAOD was shown to be 9.39 times higher (95% CI: 7.70-11.15) [18]. Additionally, SLE was found to be a separate risk factor for PAOD. This is because patients with SLE aged under 34 years had the highest adjusted risk of PAOD (HR: 47.6, 95% CI: 26.8-84.4) [18]. The risk of PAOD peaked in the first year of follow-up and then gradually dropped. As a result, SLE is a distinct risk factor for the emergence of cardiovascular disease [37].

Using the non-invasive ultrasonic biopsy score, Abu-Shakra et al. conducted a case-control study in 2008 to identify intimal and medial alterations in the common carotid and common femoral arteries of 51 patients with SLE and their matched controls [38]. These alterations are highly prognostic of the onset of clinical CVD [39]. A score of 0 means that all four blood vessels are normal, and a score of 40 means that all blood vessels have symptomatic plaques. This score goes from 0 to 40. Patients with SLE had an overall ultrasonic biopsy score 1.8 times greater than the controls [38]. Atherosclerotic plaques were much more common in patients with SLE, with an odds ratio of 3.17, and 28% of SLE patients had at least one of the four arteries affected by an atherosclerotic plaque, compared to 10% of control patients [38]. Only 37% of the SLE patients had standard scores in all vessels, as opposed to 67% of the controls [38]. In a case-control study, 65 patients with SLE (mean age: 40.3 ± 11.6 years) and 69 control subjects (mean age: 42.7 ± 12.6 years) without a history of coronary artery disease were screened for the presence of coronary artery calcification using electron-beam CT [40]. The findings of this investigation revealed that lupus patients (20 of 65 patients) had coronary artery calcification more frequently than control participants (6 of 69 subjects) (p=0.002) [40]. The mean calcification score was 68.9 ± 244.2 in patients vs. 8.8 ± 41.8 in controls (p=0.001). The study found that the age of onset is also lower in SLE and that the prevalence of coronary artery atherosclerosis is noticeably higher [40].

A recent case report by Al-Jehani et al. reported Libman-Sacks Endocarditis (LSE) in a previously undiagnosed patient [41]. Immunology tests, however, led to the diagnosis of SLE. An even rarer case, Coxiella burnetii endocarditis in a patient with SLE, was reported by Alqallaf et al. [42]. SLE's most common specific valvular involvement is LSE, with frequent involvement of the left-sided cardiac valves [43]. A cross-sectional study by Moyssakis et al. evaluated the prevalence of LSE in 342 consecutive SLE patients (297 females and 45 males) using Doppler echocardiography. LSE was diagnosed in 38 cases (24 mitral, 13 aortic, and one tricuspid valve) [44]. This study concluded that Libman-Sacks verrucous lesions were reported in one out of 10 patients suffering from SLE, and the lesions were associated with disease duration, activity, and antiphospholipid antibodies. These cases highlight the importance of endocarditis as a presenting cardiac manifestation in SLE. 

Less common cardiovascular manifestations of SLE include vasculitis and aortic dissection. Leone et al., in a comprehensive review, described that complex interactions among the vascular endothelium, inflammatory cells, cytokines, autoantibodies, and immune complexes play crucial roles in SLE-induced vasculitis [45]. Gamal et al. conducted a retrospective study on 565 SLE patients (42 males, 523 females; mean age: 32.7 ± 9.5 years; range: 13-63 years) [46]. Cutaneous vasculitis was found in 59.2%, visceral vasculitis in 34.0%, and 6.8% of total vasculitis patients. The patients with vasculitis showed a higher prevalence of hypercholesterolemia (p=0.045), diabetes mellitus (p=0.026), higher Systemic Lupus Erythematosus Disease Activity Index (SLEDAI) at disease onset (p<0.001), and Systemic Lupus International Collaborating Clinics (SLICC) Damage Index (p=0.003) scores [46]. Spontaneous coronary artery dissection (SCAD) is another uncommon cardiac SLE manifestation, with few case reports in the literature so far. A case report by Chaaban and Kshatriya described a case of SCAD in a 37-year-old female with hypertension, and a history of smoking, both risk factors for the disease [47]. Ullah et al. systematically reviewed 10 articles about SCAD associated with rheumatologic conditions and found that many cases were related to systemic lupus. Furthermore, most patients presented with non-ST elevation myocardial infarction involving the left main coronary artery [48]. SCAD is an underdiagnosed disease that requires a high index of suspicion.

## Conclusions

This systematic review studied the link between SLE and CVDs while considering the wide range of associations between these phenomena. In addition, the possible causal pathways were also explored. Our study shows a greater prevalence of a diverse range of cardiovascular diseases in SLE, which leads to increased morbidity and mortality. Furthermore, SLE is an independent risk factor for PAOD and MI. More research on the connections and associations between SLE and CVDs, including the survival rates of patients with CVDs as a sequelae of SLE. is required to improve short-, medium-, and long-term patient outcomes. This is because, even though survival rates in SLE have improved over the years, deaths due to CVDs in SLE have not shown a similar trend. Protocols should be developed to ensure that clinicians maintain a high index of suspicion when encountering patients presenting with CVDs such as PAOD, MI, pericarditis, myocarditis, or conduction defects so that they are investigated in a timely manner.
